# Critical Thinking, Formation, and Change

**DOI:** 10.3390/jintelligence11120219

**Published:** 2023-11-28

**Authors:** Carlos Saiz, Silvia F. Rivas

**Affiliations:** Department of Basic Psychology, Psychobiology and Methodology of Behavioral Sciences, University of Salamanca, 37005 Salamanca, Spain

**Keywords:** critical thinking, causal explanation, decision making, problem solving, instruction and evaluation

## Abstract

In this paper, we propose an application of critical thinking (CT) to real-world problems, taking into account personal well-being (PB) and lifelong formation (FO). First, we raise a substantial problem with CT, which is that causal explanation is of little importance in solving everyday problems. If we care about everyday problems, we must treat the identification of causal relationships as a fundamental mechanism and action as a form of solution once the origin of the problem is unequivocally known. Decision-making and problem-solving skills should be the execution of the causal explanations reached. By acting this way, we change reality and achieve our goals, which are none other than those imposed by our PB. However, to achieve changes or results, we must have these fundamental competencies in CT, and these are not innate; we must acquire and develop them, that is, we must train ourselves to have CT competencies according to the demands of today’s world. Finally, in this paper we propose a causal model that seeks to identify and test the causal relationships that exist between the different factors or variables that determine the CT-PB-FO relationship. We present some results on the relevance of causality and how to effectively form and address real-world problems from causality. However, there are still questions to be clarified that need to be investigated in future studies.

## 1. Introduction

In this special issue, we “… explore the application of CT to real-world situations and everyday life…”. The authors of this issue are asked to answer how CT is applied in our daily lives. This paper will answer this question by understanding that CT is a problem-solving process based principally on causal explanation. If we want CT to be applied to real-world problems, what it must do is solve them, and these today are more challenging and complex than they were a little more than two decades ago. To solve a problem is to achieve our goals; to do that, we need to explain reality to act most efficiently. Daily life consists of events, things that happen, and behaviors demanded by those events, which have consequences or effects. To understand reality, we need causality. For this reason, our proposal prioritizes causal explanations in the solution of the problems of daily life. Now, to achieve our goals, we need to think and act well to achieve something like personal well-being. However, the skills that make it possible to achieve our goals and, therefore, our well-being must be acquired or developed because we are not born with those skills. Thus, in CT, the skills that best address the problems of today’s challenging world must be taken into account and developed in order to achieve our goals effectively. In short, we must treat the CT-PB-FO dependency relationship to offer a way to apply CT to daily life.

We will develop these aspects in five sections. In the first section, we will deal with the limitations of CT due to its difficulty in adapting to the new times because of its lack of response to the problems of today’s world; in the second section, we will explain the changes and new demands of the present times that impose new strategies for confrontation; in the third section, we will deal with the need to improve CT given the enormous current difficulties in its acquisition, something that can only be achieved through training; in the fourth section, we will deal with what gives meaning to all this, namely, achieving a certain personal well-being; and in the fifth and last section, we will propose a causal model integrating all these aspects to achieve the change we need to achieve that personal well-being or to be able to face the problems of the real world effectively. The order in the treatment of all these sections follows a logic that imposes the dependence of one aspect on another: limitations appear with the new times, these and require continuous training to face them, and this improvement of our skills is key to achieving our welfare, and this is only achieved by changing the reality, which is only achieved from the causality. This is the story we are going to tell.

## 2. Critical Thinking: Limits and Ways to Overcome Them

Is the field of CT growing sufficiently, in the sense of evolving as much as it could? Keeping the historical and important distances, the same thing is happening to CT that happened to physics before the appearance of the geniuses of the Renaissance. Physics did not evolve as much as it could have because of, let us say, the “Aristotelian dominance”. The influence of this genius was such that it prevailed over that of another exceptional mind, Archimedes. If it had had the same influence as Aristotle’s physics, the thought of this genius in physics, engineering, astronomy, and other fields that aspired to the understanding of the world, perhaps that wonderful period of the Renaissance where science was invented would have happened long before the intellectual darkness of the Middle Ages (Wootton 2015; to understand the analogy with Toulmin and the consequences of Aristotle’s greater influence than Archimedes on physics and the subsequent development of science, see his excellent analysis of Wootton 2015 and the development of the history of science in Flores 1979). Drawing an analogy with the development of physics, we can say the CT has also experienced a similar influence, the “Toulminian dominance” (Saiz 2020). Toulmin (1958, 2003), with his excellent work on the use of argumentation, determined the development of CT, and it has been very difficult to escape from that model (see Baron 2024; we must make it clear that everyone recognizes Toulmin’s fundamental contribution to stimulating the development and importance of CT). As with physics, logic and argumentation largely guided the evolution of the CT field. Again, the predominance of rationalism leaves little room for empiricism, and this is a problem because both should have equal prominence in science. Moreover, when applying CT to everyday life, we must act or intervene in reality. For this, a good reflection is not enough; this is materialized by employing decision-making and problem-solving strategies. However, these new strategies or thinking processes result from research in cognitive sciences starting in the 1990s (Holyoak and Morrison 2005; Sternberg and Funke 2019). This new knowledge involved incorporating processes different from argumentation (pre-decisional), such as those related to problem-solving and decision-making (post-decisional).

The fact of incorporating post-decisional mechanisms generates conceptual difficulties because they are processes of a different nature from argumentation. In fact, by contemplating these new competencies, the homogeneity of the prevailing theory of argumentation is broken, since skills are introduced that have more to do with what would be a theory of action (Rivas and Saiz 2023; Saiz and Rivas 2011). This causes important theoretical problems due to the resistance generated by the acceptance of these mental processes as belonging to the field of CT (Johnson and Hamby 2015). It is difficult to integrate both kinds of processes: the representational ones and those that execute them. These discrepancies are also at the origin, let us say, of a certain paralysis in the evolution of the field of CT because it has delayed the incorporation and application of these new mechanisms (Rivas and Saiz 2023).

Toulmin’s model has established argumentation as the main actor of CT or as the competence that determines and conditions everything; the significant philosophical influence on CT, we believe, must remain but in another way. In our opinion, this domain must be the guide of correct reflection, but it must not determine other things simply because it cannot. Argumentation allows us to represent in the best possible way the reality of that part of the world we need to understand or comprehend; however, in most cases, it is necessary to apply, to execute those conclusions we have reached in that representation. However, at this point, the skills we manifest are very different from everything that has to do with reflection; in other words, action is the daughter of thought (Franco and Saiz 2020; Marcio 2001). Its concretion consists of interacting with the world through behavior, which has consequences that must be explained to give meaning to events, but this understanding or reflection is not enough; we need causality. We want to clarify that we are referring to *causal explanation and its demonstration*; causal identification alone is insufficient, and verification is also needed. This idea is equivalent to what Bird (2010) calls “Holmesian inference” (for a more in-depth analysis, see Azar 2019; Grimm 2010; Guamanga et al. 2023). For us, this is the unseen elephant in the CT room (Saiz 2020).

Those CT approaches based on argumentation also consider explanation or causal reasoning but from a different epistemological approach. The explanation of behavior can be approached from comprehension or explanation. In the first case, we seek to understand behavior from intentionality (teleological approach; see von Wright 1979); in the second, we seek to understand behavior from causal explanation (see Toulmin 1977; von Wright 1979). Our position is clear: CT can only progress and answer everyday problems from *causality or causal explanation*. The reason for our position is obvious: intentionality is unobservable.

This will be the central thesis of our work. From here, we will justify that, without this approach, CT cannot progress because its main problem lies in being characterized by too much representation and too little action; the predominance of the pre-decisional and the merely testimonial character of the post-decisional are important limitations. In the present paper, we will offer a solution to this problem with the help of Judea Pearl’s *new science of causality* (Pearl 2009; Pearl and Mackenzie 2018).

As we said, if the mental representation resulting from reflection was enough to survive, we would not need to translate it into reality. Translating our ideas into the world forces us to act; it requires action and behavior. For different reasons, these competencies have never been well integrated within primarily argumentation-based approaches to CT. They have not been well integrated because the incorporation of causality and skills such as decision making and problem solving remain disproportionately minor compared to the basic forms of formal and informal reasoning; we need only go through the content and space occupied by formal and informal argumentation in relevant works in the field (see Bassham et al. 2023; Bowell and Kemp 2002; Epstein 2006; Fisher 2011; Freely and Steinberg 2013; Foresman et al. 2017; Govier 2014; Jackson and Newberry 2012; Johnson 2000; Johnson and Hamby 2015; Kenyon 2008; Moore and Parker 2021; Tittle 2011; van Eemeren et al. 2007; Walton 2006; Walton et al. 2008). However, other CT perspectives did understand the relevance of post-decisional mechanisms following substantial contributions from cognitive science (see Ennis 1996; Facione 1990; Halpern 1989; Paul 1995). These decisive early contributions have given way to perspectives that are more comprehensive and better define what CT is today, in our view (see Baron 2024; Dwyer 2017; Facione 2011; Facione and Gittens 2013; Halpern and Dunn 2023; Hunter 2014; Paul and Elder 2012; Pinker 2021; Saiz 2017, 2020; Sternberg 2021; Sternberg and Halpern 2020; Sternberg and Funke 2019). However, despite this important progress, there is a need to reconceptualize the CT approach from causality. As we pointed out, this would be one way to solve the problem of slower CT progress, but only in part. It is not as important to better integrate the fundamental cognitive competencies and change their function as it is to put causal explanation at the center of CT; this would be one part of the solution to the problem. The other important part of the problem would be the scarce attention given to behavior, action, and change in the different approaches to CT (Saiz 2020). If CT wants to offer effective strategies for adaptation to the real world, it is reasonable to consider the new realities. It would be an anticipated failure if CT did not consider current changes and demands because the world of the 21st century demands greater interaction, communication, group decision making, and solutions to new problems. However, all these demands can hardly be attended to if we do not opt for causality as the main actor in the proposals for improving daily life with CT. Let us see in what sense this does not seem to have enough influence.

## 3. Critical Thinking: The Changes and New Demands of Today’s World

The changes in the world of this century are dizzying and impose the challenge of new demands. For our purposes, two key dates have marked and will mark these transformations: 2007 and 2023. In 2007, the first mature smartphone (iPhone) appeared, enabling the proliferation of social networks in the following years. In 2023, the first mature global artificial intelligence (AI) projects appeared, and in recent months, they have developed in such a way that expectations soar thanks to the *OpenAI* project (ChatGPT). In a short time, the advances in AI and its combination with neuroscience (Hawkins 2021) have been spectacular and worrying. Adaptation is, therefore, a difficult task.

To support this pessimistic statement, we present and discuss data that leave no doubt about these deficiencies (see Saiz et al. 2020). Additionally, there are very convincing data from attention experiments. Gloria Mark notes that today, we can only pay attention or concentrate for a maximum of 47 s, whereas 20 years ago, we could maintain our attention for up to two and a half minutes (Mark 2023). Also, we can see these limits by getting into technical and complex work on biases and noise by Kahneman et al. (2021).

There is a principle called the *universal law of learning* (ULL), which is that any person, institution, or society has to learn at least at the same speed with which the environment changes to survive and, if it wants to progress, it must do it faster (Marina and Rambaud 2018). The question is, in general, do we survive or do we progress? In particular, in CT, the same question is posed; our answer is that we will certainly not progress. Recall that the title of this work consists of three terms, and the second is included in the idea of ULL, *learning* to survive or progress. This process is the one that allows us to understand our interaction with the world better because what we need is to adapt, and learning is what makes that possible (Sternberg 2021); if we do not learn, we do not survive. Vygotsky’s (1993, 1978) idea of intelligent adaptation, which according to him depends on the learning capacity we possess, is recovered.

Overstimulation causes concentration and attention problems. If attention deteriorates, our thinking can only be superficial, so deep or complex thinking becomes an exception. Observation of our students in recent years allows us to state that they are not able to make more than two inferences in a row in their daily academic work. A relevant and solid conclusion needs at least three inferences; therefore, deep thinking is called into question. Increased entertainment causes a greater predominance of MINIMAX—the law of MINIMUM effort and MAXIMUM gratification—(Saiz et al. 2020). This law is adaptive from the species’ point of view, but not in many other ways. Well-founded and contrasted knowledge does not come easily; it requires good observation, effort, and deep thinking. Contrasting or evaluating a position, thesis, or conclusion requires the application of the appropriate criteria, which are the result of reflection or good judgment. This ensures the credibility of these ideas or knowledge.

Attention and concentration problems make the acquisition of knowledge (or learning) difficult. Knowledge is inferential; an idea or concept is the conclusion or result of a reflection, which requires relating information to categorize or establish relationships of belonging or class. Knowledge ends when we can causally explain reality and modify it, not before; only then, we can speak of the product of that process, namely, the result of the acquisition of knowledge based on a causal explanation. We agree with Perkins (2009) when he states that we can only say that we know something if we can apply it.

Cognitive problems resulting from these new times cause us deficiencies that are not found in our genes. However, they are not the only deficiencies that beset us. We all know that our cognitive system is not perfect. These deficiencies, biases, or intellectual limitations, which are the fruit of descriptive research in thought, have been known for decades. Baron (2024) rightly distinguished research in thinking as descriptive, normative, and prescriptive. The first descriptive works, i.e., those aimed at finding out how we think in everyday life, already revealed the lack of logic in our thought processes (Henle 1962). However, neither deficiencies of one type nor the other figure prominently in CT development and improvement initiatives, despite their clearly applied nature (Saiz 2017).

At least in our country, what we have observed in recent years among university students is a significant increase in family and social protection and permissiveness. Greater protection or overprotection reduces personal autonomy, and permissiveness or consent reduces personal responsibility. If the level of demand is low, MINIMAX nullifies initiative and the search for solutions since there will already be someone to do it for us. On the other hand, if behaviors have no consequences, the essential learning that our actions provide us with disappears due to the lack of responsibility, since someone else will assume the consequences in our place.

For our work, the development or improvement of CT is a difficult task to perform when personal initiative has deteriorated and the consequences of our actions are not assumed individually. Therefore, if we are looking for CT to be a good guide in our daily or everyday life, we must take into account the cognitive and behavioral problems caused by the social changes that have occurred in recent times; unfortunately, these considerations are rather scarce in most of the initiatives aimed at such improvement (Saiz 2017).

Resnick (1999) said some time ago that the ultimate goal of education is thinking, although she did not have CT in mind. Today it is the object of desire of education, in fact, the desired result of education (Dwyer 2023). When we talk about thinking, CT, or intelligence, we know that we are referring to higher-order cognitive processes and different models of mental functioning. A classical model of intelligence based on IQ is a good predictor of academic or job performance, but it does not predict as well the performance in the face of everyday problems or real-world problems (Halpern and Butler 2018; Halpern and Dunn 2021; Rivas et al. 2023b). The complexity of today’s world problems is better coped with by other models such as some CT approaches, which incorporate skills such as problem-solving or decision-making strategies (Halpern and Dunn 2021). Other models of intelligence equally cope well with everyday problems. Robert Sternberg, a relevant representative of the theories on intelligence, has in recent years put forward an integrative approach based on the classic concept of adaptation and learning to solve problems. Expressions such as “learning to think critically”, “adaptive intelligence”, or “successful intelligence” (Bonney and Sternberg 2011; Sternberg 2018, 2021) are conceptions that are difficult to distinguish from what many understand as CT. Sternberg (2021) himself differentiates general intelligence and adaptive intelligence, and we believe this is a good distinction; we understand intelligence as the potentiality (Ackerman 2018), which we cannot know or measure, and the expression of that potentiality, which we can know, measure, and improve. Thought processes are such expression and are the cognitive components of CT (Saiz 2020). In short, thanks to these skills, we reach our goals, solve problems, or change the situation.

Sometimes we forget that our cognitive system is at the service of our biological nature, ultimately survival, for which we need adaptation to the environment. The question, for example, of why we think has a very simple answer: because we need to, or because want something we do not possess and want to get it, or we want to avoid something we do not want; in all cases, we have a problem to solve. We return to our approach at the beginning, that is, the fundamental goal of CT is to resolve to achieve change, to act to achieve our purposes; the front and reverse of our cognitive coin are thought and action.

We said that CT is the object of desire, not only of education but also of companies and different organizations of different natures (Dwyer 2023; Halpern and Dunn 2021), at least in words. The expression “we must think critically” is more of a mantra, nothing more. Teaching or learning to think well starts with the difficulty of knowing what it is to think well or critically and what it is to teach or learn. Necessity sharpens the mind, and the pandemic catastrophe and other global misfortunes have contributed to this, in the sense of becoming aware of the increasingly complex and sophisticated problems of our world. In part, this has been a stimulus to more frequently orient and define CT as a set of cognitive skills that enable us to obtain desired results or to solve problems in the most effective way. More than half a century ago, Newell and Simon (1972) were already pointing the way. We can finally say that we are emerging from the “Toulmian dominance”.

CT is increasingly understood as a matter of solving problems, which requires action. For this reason, we go a step further and say that to *think critically is to reach the best explanation for a fact, phenomenon, or problem to know or to solve it effectively* (Saiz 2020, p. 27). As we have proposed above and will develop further on, we incorporate explanation, and causality, because efficacy is not possible without it. We give the mechanism of causal explanation the maximum protagonism, but it needs the collaboration of argumentation (and not the other way around) to decide and solve. The solution is already in the causal explanation, we only need to act to produce a change and achieve our goals effectively. Argue to help explain, explain to help decide or solve, and solve to bring about change effectively. For us, these are the fundamental skills of CT and the relationship that is established between them (see Saiz 2020). These are the cognitive components of CT, but there are others of a non-cognitive nature, such as dispositional, motivational, attitudinal, or metacognitive, without which CT cannot occur. Some directly integrate these two dimensions, cognitive and non-cognitive, defining CT as a metacognitive process (Dwyer 2017, 2023). We believe that this is neither a good idea theoretically nor practically. Conceptually, we increase the confusion, since there are already enough problems with metacognition and motivation (or vice versa), at least in one of its meanings, that is, regarding the planning and organization of behavior. Thus, we practically tie our hands from the point of view of instruction or improvement of CT (Rivas et al. 2022). Awareness of what happens mentally is always present in any CT improvement initiative; without the “awareness of,” improvement is not possible, and there can be no learning or acquisition without that level of awareness or metacognition. The problem lies in how to modify or promote metacognition and know that we have done it; we are facing the same problem we have with motivation: its manipulation or operationalization. Our eternal wall is the mental and its quantification.

## 4. Critical Thinking and Formation

Teaching or learning to think critically, for us, consists of developing those fundamental skills mentioned above. At least, *what to* teach or learn we have detailed; without knowing what we are talking about, there is little we can do regarding CT development. Diane Halpern has justified this very well in different relevant works, stating that to change or improve CT, the skills that are intended to be improved must be concretely specified (Halpern 1998; Halpern and Dunn 2023; Marin and Halpern 2011; Saiz 2017). However, do *we teach, learn, or form ourselves?* What are the differences? Alternatively, are we talking about the same thing using different terms? Before the internet existed, it made sense to talk about *teaching* where the administration or organization of knowledge and the transmission of knowledge were the fundamental tasks of education. The fundamental activity was the reception and reproduction of the content, with a predominance of declarative knowledge and, consequently, little practice and application. The teacher–student relationship was unidirectional. In the post-internet era and until the end of the first decade of this century, education is now understood more as *learning*. The acquisition process is now focused on understanding, and reception or passivity is being replaced by interaction through questions, developing synthesis and relating content. Practical and applied activities are also beginning to be incorporated. Procedural knowledge begins to gain prominence, as well as learning management (*learnability*) as opposed to knowledge administration. Finally, the teacher–student relationship is bidirectional (see Saiz et al. 2020).

What is happening today? Our experience is that in university education in our country (although again, we believe it is generalizable to other places), the development of critical thinking is not happening, even though it is the time and place where it could and should happen. There are somewhat concerning studies that highlight that university students, as they progress through their degree, worsen their level of thinking (Arum and Roksa 2011). In contrast, our students, because they go through our instructional program in the first year of university, not only maintain but improve their CT level four years later (Rivas and Saiz 2016). We only want to highlight a general way of CT development; we have specific techniques for its improvement, verified in several studies.

**Figure 1:**
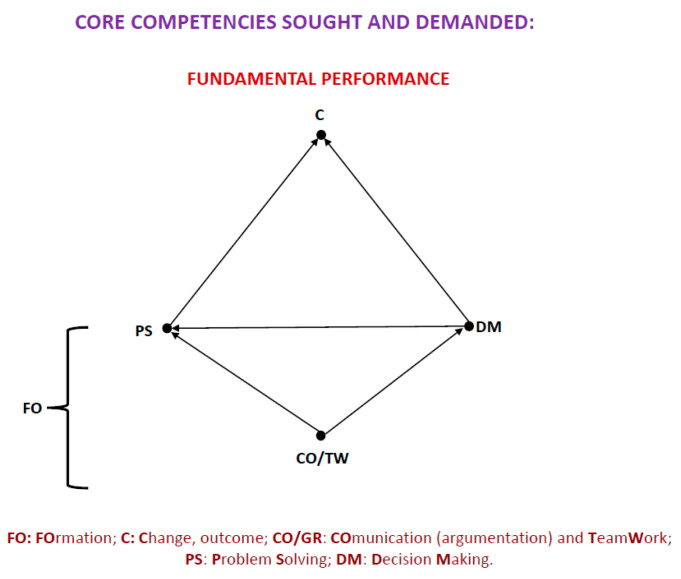
Core competencies sought and in demanded.

We have been working for some time on *formation*, rather than learning or, of course, teaching. In our opinion, the consequences of the changes and new demands of the present times force us to use a new approach to manage these times of great uncertainty. Companies have long been demanding the same skills, regardless of qualification: communication skills (argumentation), teamwork, decision making, and problem solving. How curious are they asking us for professionals with good CT skills (Saiz et al. 2020)? (See Figure 1 two pages later) In reality, they are asking for much more because it is no longer enough to have a good grasp on our own domain; we also need to be able to solve new problems using our own preparation or expertise. In the workplace, horizontal or transversal competencies are required to be effective, not only efficient.

These demands from the world of work are a logical consequence of the great mobility and flexibility of the market and business. Studies have shown that, for some time now, graduates will change jobs between twelve and fourteen times during their working lives (30–35 years), and this mobility seems to be increasing. What will be increasingly in demand are what they call “knowledge nomads” (also known as *knowmads*); see Saiz et al. (2020). If this is the trend, how can companies not need professionals with horizontal competencies such as those of CT? The question is, is technical and personal formation going in that direction? The answer is clearly no. To the question of what to do, the answer, for us, is *formation*. Let’s see how.

Today, society at large is absorbed in smartphones, social networks, YouTube, and such … and classrooms are colonized by this obsession. Moreover, AI is rapidly advancing in the performance of tasks. Educational institutions are beginning to be replaced by tech giants (*GAFAM*). A study done in the USA shows that three of these five technological companies have discovered the big business of training and are investing around 4 billion dollars; this is equivalent to three times the GDP of our country (see Marina 2017, 2022a). *STEM* is the big professional demand and will remain so for quite some time. The majority of American universities seem to have experienced a kind of abandonment of CT, progressively replacing it with identity politics (identitary thinking; see Marina 2017, 2022a). Who is going to be formed in CT? Clearly, the “immunity” that the CT vaccine can provide will not be reached; instead, a vulnerability to disinformation, the undervaluing of science, the devaluation of truth, the difficulty in selecting information, or an overall lack of direction may become pervasive. Faced with this prospect, what is the plan? It is clear to us that the current teaching and learning models are insufficient.

Given the present circumstances, *formation* seems to be the only solution. Knowledge based on causal explanation should be the standard acquisition process; when possible, work must rely on the social support of a community of inquiry. The formation process should be based on solving real problems and seeing if changes have occurred or results have been achieved. Likewise, asking questions remains a fundamental process, as is mainly procedural work, with inter- and intradomain practices and applications to promote generalization or transfer. Formation must be permanent, and the autonomy and the initiative of the one who forms must also be constant (see Saiz et al. 2020). Not without pessimism, we can rescue the old slogan of *do it yourself* because deepening and mastering fundamental CT skills and applying them effectively to everyday life requires this kind of preparation. In the current circumstances, the model of autonomous and lifelong formation is the only preparation model that can slow down the negative effects of the ULL, the one that can fully develop acquisition processes and reach the maximum availability of horizontal competencies that are in demand. This approach retains some of the features of the other two, education and learning, where relevant, e.g., the lecture, and amplifies the use of those features it shares. For instance, answering questions should be used in most of the formation process, we would say in 85–90%. However, this approach only provides the framework for CT development. How to operate in this context requires specific methods of action based on those we have already contrasted (see Rivas and Saiz 2023; Saiz and Rivas 2011, 2012, 2016; Saiz et al. 2015).

## 5. Critical Thinking and Personal Well-Being

As we said, the development or improvement of CT demands a what and a how—what to improve and how to achieve it—but also a why or what for. We think because we need to; specifically, we think critically to solve real-world problems that affect us directly or indirectly. From this way of looking at CT, we have taken an important step by placing action at the core of this approach. It is in the interaction with the environment that we solve problems or achieve our goals. In this way, CT begins to make some sense, were it not for the fact that we forget something essential: why solve problems? Because we need it? This is saying rather little. What needs are we talking about? Here, we enter the realm of the non-cognitive dimension of CT. Motivation to solve our problems would be a first approximation to what it is that drives CT skills to get going.

In reality, the ultimate goal of the human being, some would say, would be happiness, but we know that this idea is too polysemic (Marina 2022b); it is a fuzzy concept that is difficult to use. However, most of us would agree that something like happiness, personal well-being, or quality of life could reasonably fit the idea of that ultimate motivation of the human being, which would be responsible for our personal fulfillment. Flanagan et al. (2023) discuss in their work these elusive concepts from several points of view, making us doubt the very title of the book: *Against Happiness*. For the purpose of this paper, we propose that the ultimate reason for solving problems is achieving an acceptable personal well-being. Globally, the concept of well-being that interests us is that in which the person values that his or her life has meaning (see Flanagan et al. 2023). We are always considering this idea applicable to the adult population. The way to measure this meaning of personal life is with a scale or with situations in which there would be no behavior or it would be of a certain form.

In the present project, personal well-being is a feeling of satisfaction that comes from having achieved economic and emotional independence, which allows the person to achieve the goals he or she sets for him or herself while at the same time perceiving that his or her life has meaning. Let’s say that it is a mixture of personal and social achievement with vital meaning. The way in which we seek to measure this personal well-being is by observing the behavior, in terms of their functioning at work and in their personal environment, and the absence of conflict or major problems that allow a calm and meaningful development in their daily life. In short, the person feels or experiences that he/she can manage his/her life and that it has meaning. Thus, achievements and problem solving provide a sense of life control that offer peace of mind and satisfaction (Flanagan et al. 2023; Marina 2022b). From the point of view of measuring personal well-being, we care about what we observe, that is, personal and social performance. However, we must recognize that we are at a very early stage in assessing personal well-being, as we still need to operationalize this scale. An important reason for this difficulty is that we avoid using self-report measures and seek to use observations or behavioral data. However, once we can move in this direction, we are using Ryff’s scale (Díaz et al. 2006; Ryff and Keyes 1995).

The expression *knowledge begins with the wanting* captures very well the origin of knowledge. To know or to seek knowledge, we must have the *will to want* and the *desire to want*. The motivational and the emotional are inseparable from cognition and are what make us move or act; there is no adaptation without will and feeling (old concepts, today modern, already used in Greek anthropology by the Sophists and developed by Socrates; see Flores 1979). It would be difficult to imagine anyone, except by pathology, who would not *want* (in its double sense) *to* achieve a certain personal well-being. Therefore, we could say that this is our ultimate end or most cherished goal, and to achieve it, we must overcome the obstacles or solve the problems that arise throughout our life cycle. Now we have given meaning to the activity that we believe best integrates the different CT competencies, i.e., problem solving. But this forces us to understand, in turn, what problems we are talking about or whether there is any kind of problem that guarantees personal well-being, once they are solved, and the answer is yes (Saiz 2021). We can affirm that there are two general classes of problems linked to the dual nature of human beings: the biological and the social. As s living organisms, people must find a way to sustain themselves to survive, and as beings dependent on others, they need the group to live and progress. Consequently, the important problems that any person will always have are of only two types: *professional and personal*. If a person has a profession in line with his or her qualifications and desires and has a supportive social network, then he or she is in a position to achieve the desired personal well-being (see Saiz and Rivas 2020).

It is too often forgotten that reaching this goal, the ultimate meaning of survival and living, occurs in a critical period of our life cycle. It is not until the age of eighteen that society considers us to be full adults, that is, responsible for our actions. Society asks us to move seamlessly, overnight, from the stage of almost adolescence to full-fledged adults. In reality, it is at this point in the life cycle that our most exciting and interesting, but also critical, period begins. From the age of 18 to approximately 30-35, we must achieve the two essential objectives for every person: *economic independence and emotional independence or personal maturity*. The first is the necessary condition for that personal well-being, and the second is its sufficient condition. In this critical period of 10–15 years or so, we must achieve material solvency and personal balance to be able to go through the rest of our life cycle without too many shocks or insurmountable difficulties. The *diachronic dimension* is rarely taken into account when dealing with problems, nor is the fact of how transcendental this critical period is for the rest of the life cycle. Moreover, it is worth noting that this stage of these 10–15 years coincides with the period of higher education or professional formation studies, which offer access to the professional world in the best conditions (see Saiz 2021; Saiz and Rivas 2020).

This critical period of preparation for a professional future also coincides with the most intense years of our social development. Moreover, formative and personal experiences occur temporarily together, with the corresponding interactions and influences that will continue to occur until the end of our life cycle. These influences should make us aware of the importance of the professional aspect of our lives in today’s world; moreover, the increased level of demands and professional burden today interferes with and conditions our personal lives excessively. Due to this pressure to be productive, it is often very difficult to distinguish between professional and personal problems. However, the good thing about this is that the competencies or skills that are demanded of us professionally and that we mentioned earlier are those that also serve us for personal problems. New roles and sophistication in both personal and professional relationships require good decision-making and problem-solving strategies, in short, good coping strategies (see Figure 1).

Figure 1 shows the core competencies required (i.e., a high level of CT is requested) to achieve results or produce changes, both professionally and personally. If these changes go in the desired direction, professional and personal progress guarantee our personal well-being. Let us note that this figure is represented by a diagram with nodes and arrows, which establish relationships that aspire to be causal relationships with the approval of facts. These diagrams are not only a system of didactic representation; they are a logical formulation that intends to concretize the ideas and their relationships so that they can be verified. They are representations that can be checked. To achieve this operationalization, this system of diagrams is based on graph theory, which is a logical-mathematical system (Wilson 1983) that provides us with the necessary precision to be able to develop the proposals we make of causal models and to able to validate them (Pearl et al. 2016). We will detail the significance of the elements of these diagrams later; for now, it is enough to see the direction of the arrows in terms of what determines each thing. We only want to illustrate the role of the key competencies of CT and the relationships between them.

**Figure 2:**
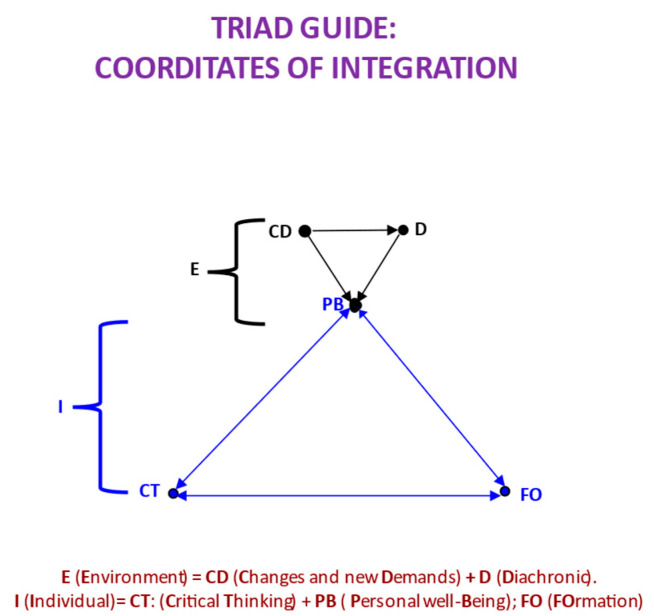
Integration of environmental and personal determinants of personal well-being.

A good part of what has been explained so far is shown in Figure 1. Competencies, training, and change or achievements are included. The competencies represented in Figure 1 are those demanded professionally, the formation that allows us to develop the CT, which makes it possible to achieve our goals, what are the desired change, and this provides us with subsequent personal well-being. But the good or the best possible results, let us not forget, have an end: our personal well-being. Therefore, we must integrate them with the rest of the determinants discussed so far to better understand the relationship between CT and personal well-being (see Figure 2).

In Figure 2, we can find all the determinants of personal well-being that we have analyzed so far and represented with the logical precision of a graph model as a proposal for a causal model to be further specified and demonstrated. In the following section, we will incorporate the factors that summarize each node here and that do not appear in Figure 2. In addition, we will describe some data that support the model, only a few, because this is a proposal for future studies. In this figure, on the one hand, we have the environmental causes that influence the diachronic dimension in the different stages of the life cycle, especially in the critical period already described. The causal relationship of these factors is only partially clear, as we will detail later, due to the types of connection between nodes. For the time being, we will only deal with the conceptual description. Personal well-being depends to a certain degree on these environmental factors, which must be quantified. On the other hand, we see personal causes, CT competencies, and the formation needed to develop it. Again, these causal relationships are not clear, in this case, because of those double arrows that appear. When achieved, causality is always expressed with a unidirectional arrow. Later on, we will further clarify these logical and causal precisions. For now, let us stop at the representation of the diagram, where the environmental and personal lead to the change that makes it possible to achieve well-being. The way to achieve this change is to decide and effectively solve these new challenges, acting based on the explanation that allows us to know the causes of the events to modify or adapt them. However, all this is only possible with the good development of CT, that is, with a good formation.

## 6. Critical Thinking and Change

The purpose of what has been discussed so far, let us remember, is to justify a proposal in which CT and its development are the best way to effectively solve real-world problems based on causal explanation and action. The identification of the causes of a problem is the diagnosis; the plan of action, the treatment, and the changes produced, this is, the elimination of the problem or the cure. In this section, we will develop our proposal for diagnosis, treatment, and solution, and finally, we will propose a verification methodology for an integral causal model.

At the beginning of this paper, we spoke metaphorically of the “Toulminian dominance” to raise a problem in many of the CT approaches. If we were to review the books cited (and others that we have omitted) as examples of the predominance of Toulmin’s model, we would find the following, with minor variations. All these publications always deal with argumentation, its structure, and evaluation, the two general forms of reasoning (deduction and induction), fallacies, calculation of probabilities, legal, moral, and sometimes aesthetic reasoning, rhetoric, and some treatment of new technologies and pseudoscience. Causal reasoning and its verification (hypothetical reasoning) usually occupy a section that never has the extent of either argumentation or deduction; it is a small part of induction. The way to acquire these skills rests basically on conceptual understanding and practicing through specific exercises for each type of reasoning, with minimal application to the real world and everyday problems. Let us say that a model of teaching and, in part, of learning, as described above, is followed.

The question that arises is the following: after all the changes that have occurred so far this century—the ubiquity of screens, social networks, continuous entertainment, GAFAM, STEM, AI, neuroscience—do we still believe that a CT model like this one can serve as an answer to real-world problems? Clearly not. Let’s give an example by way of analogy. As far as we know, mathematics in secondary school, in most of the countries around us, on the one hand, has always been a subject that is not understood, not seen as useful, does not raise interest, and has a high failure rate. On the other hand, this is understandable, since it is a difficult subject due to its high level of abstraction, something that has never been an easy matter. Only two decades ago, when the world was very different, the specialization routes that secondary school students chose (at 15–16 years of age) to be able to later opt for the study of certain university careers rarely included the ones involving mathematics. At that time, there were very few university students in mathematics; nowadays, the demand exceeds the capacity of these centers. What has changed? Is mathematics now easy or fun? No, but they have proven to be necessary to solve current problems that did not exist before. They are capable of providing solutions to very different fields of science; they have become transversal or horizontal competencies. Moreover, this domain of mathematics is in high demand by most companies, just like the rest of STEM. Argumentation alone cannot provide answers to the problems of daily life.

For us, this is a paradox; argumentation without action becomes useless, but if we incorporate behavior, argumentation has to be at the service of causal explanation, otherwise, argumentation and action will remain of little use. A change in the role of the protagonist and secondary actors in this CT story is needed. Fortunately, after the advances made in the cognitive sciences, new ways of understanding CT have emerged that overcome this important limitation, as already discussed. The fact of fundamentally orienting CT as a problem-solving activity makes possible not only the survival of the field but its progress too. These new ways of understanding CT are completely correct in placing the ability to solve problems as the main actor. The importance of judgment and reflection is maintained, but the ultimate goal is to provide answers to existing difficulties or problems of daily life. By endowing CT with propositivity, the action of post-decisional competencies is incorporated, and the person is faced with the need to interact with the environment, to adapt, and to try to progress.

This is the right path, but the protagonist who allows the best solutions or effectively solves the problems is still missing. The explanation, the causality, is still taken into account as a secondary actor. The causal explanation is the one that allows for solving problems effectively, not only efficiently; therefore, it cannot be a marginal actor because it is the one that tells us how to act to resolve. The same thing that happened to physics is happening to CT.

CT has made a great leap in understanding that we must be able to solve real-world problems, but it has not been consolidated because causality is not the main guide in problem solving, and neither has it combined logic and causality, as happened to physics. It begins to be decisive when science is invented and consolidated by combining two great achievements, the system of formal logic and the discovery of the possibility of finding causal relationships through systematic experimentation (see Wootton 2015). This is what CT still lacks, like physics before the Renaissance: to unite logic and causality, even though geniuses of the 19th and early 20th centuries have already shown us the way, such as John Stuart Mill, Charles Sanders Peirce, and Conan Doyle. The latter defined better than anyone else the path that CT should take and has not yet taken when he put in the mouth of his most famous creation—Sherlock Holmes—the following: “… *there should be no combination of events for which human intelligence cannot conceive an explanation”* (The Valley of Fear; Conan Doyle 2009, p. 706). This genius, in his first great novel *A Study in Scarlet* (Conan Doyle 2009), details his method of inquiry for the first and only time. For us, within the “canon” of Conan Doyle, this novel has special importance because it is the clearest and most explicit paradigm of what we are concerned with and propose: *observation, logic, and explanation*.

If what matters to us is dealing with real-world problems, *observation* is essential, but it is a skill we have barely developed, and today, with screens, it is much impaired (let us remember what has already been said, the 47 s, the maximum time of attention of which we are capable, experimentally verified by Gloria Mark). If observation fails, and it does so too often, there is little else that can be done. *Deduction* gives us unequivocal conclusions, and the observed will either support them or not. In reality, correct deductions can be made from the facts, and we can be right about what has produced them as long as we find neither counterexamples nor additional data that falsify such deductions; in this case, we will have a unique and certain explanation within a context or problem situation. The logical principles or rules of causality (sufficient necessary condition, SC-NC), which are structurally similar, are very powerful machinery if we let the facts be the ultimate judges. The in-depth description of the fundamental logical principles for proving causality and their structural similarity to the rules of SC and NC are especially technical, and a special section would be needed for all this. The interested reader can find this full development in Saiz (2020).

Observation requires a lot of practice because it is contaminated by our previous schemes and by the set of biases and distortions that, as we said before, mentally come to us naturally. From the point of view of instruction or formation, developing this skill takes much more time and effort than we might expect. The reasons for this difficulty lie in the fact that we think that to observe is to perceive well, but this is not correct. This can be better understood if we understand that the relevant facts are never the perceptually noticeable or striking ones, except by chance. The relevant facts are those that fit our hypotheses, and not the other way around. Facts become relevant or irrelevant only when we have a story or causal scenario that can give them an initial sense, when we can explain or make sense of the events. This is perhaps better understood if we keep in mind that to make sense is to know to a certain degree, and knowledge is inferential, not perceptual, at the level of processing that we operate (for a complete development, see again Saiz 2020).

The combination of observation and deduction to reach an explanatory hypothesis can already be found in Edgar Allan Poe, with his famous figure Dupin^1^ (Poe 1988). However, formally it was born with Peirce (Sebeok and Umiker-Sebeok 1979), who developed it within what he called abductive logic. However, for a long time, Peirce’s abduction has not been given attention because logicians have been very focused on deduction. They have only begun to give importance to abduction when it has begun to have importance for the theory of science, for the discovery and evaluation of scientific hypotheses, and in the first steps of AI for medical diagnosis. Today, it has already captured the interest of cognitive science and new AI (see Magnani 2001, 2009). Are all these fundamental developments of our time being taken into account in CT? We are not aware of them. However, our interest here in abduction is purely its application, as its formulation and epistemological treatment exceed our objectives (an extensive formal investigation can be found in Aliseda 2006, 2014). From an applied perspective, it is observation or facts that consume most of the work in causal explanation, and the rest is employed in deduction. Nowadays, observation and deduction are inseparable; algorithms alone are not enough if we really want to face everyday problems (again, this technical development can be found in full in Saiz 2020). Today, there is a fever for using chess as a didactic tool; however, Allan Poe already pointed out the limitation of this algorithmic game as a model for the development of intelligence because according to him, what is needed is an uncertain game that forces us to observe, like poker (Poe 1988).

We usually tell our students that CT is 80% “look, look, and look again” and 20% deduction to make them realize how difficult it is to capture the really relevant facts. As we know, the most powerful enemy of our mind is *confirmatory bias*; data that are congruent with our ideas are the ones that catch our attention, and incongruent data hardly get it. The fact that our cognitive system is essentially inductive by adaptation and conservation has much to do with the powerful influence of this bias. Adaptation to the environment to survive creates in us a very strong need to always have some explanation of the events or problems that affect us; this need makes us make sense soon and always of what matters to us, even if we are hardly sure of it. For this reason, we seek or force the facts to fit anyway. Of course, the confirmatory bias is also affected by this need.

These are two of the ten capital sins of a cognitive nature that come to us genetically (see Saiz 2020, 2022) and impede us from thinking critically or problem solving. Continuing with the limitations of our mind, we must point out naivety, or thinking that the world is fair in the face of what logic tells us—namely that the world is not fair, it just is. This belief leads us to accept ideas or reflections lacking any basis or solidity. We can see here how the dispositional (the non-cognitive) contaminates correct thinking; this happens in part because words possess a great power of seduction due to our social nature, which causes facts to be substituted for them. Another major deficiency in problem solving comes from our insensitivity to the probable. We too often confuse the possible with the probable, and this makes the solution space very large, paralyzing almost every decision or solution strategy. Every problem depends on a context that limits the options since there are general options that do not fit in a given context, and thus to consider them is to subscribe to the failure of the solution.

We have only mentioned some of these 10 deficiencies or capital sins, but this is not the end of our mental difficulties because we can also say that we suffer from what we have called “the 10 false virtues” (see Saiz 2020, 2022). The first is believing that we have mastered correct reasoning, when Henle (1962) showed us long ago that we only handle a couple of logical principles. Another of the serious limitations we suffer from is to confuse what is true with what is correct. During reasoning, when all statements are true, it no longer matters what is concluded because we will take it as valid. Thirdly, it has long been known that all things that happen spatially or temporally together tend to be considered related, even though they may or may not be. This cognitive distortion is notably guilty of confusing correlation with causation and also of attending only to presence-presence data when we want to establish contingency relationships, as we leave out the other three kinds of data (Smedslund 1997).

Finally, for us, there are three particularly serious deficiencies that prevent us from getting a good causal explanation right. To make sense of things, we need to imagine or mentally simulate events, and we do this very well, but what we fail to do is to causally simulate these situations. Constructing causal scenarios is difficult and requires a lot of practice. Constructing a causally consistent story is key to achieving a good causal explanation. However, this is not easily achieved because it is hampered by two other important limitations: on the one hand, the enormous difficulty we have in making complete sense of events or problems since we only do so partially, and on the other hand, our deficient use of counterfactual thinking. Without the imagination of counterfactual events, we are very limited in our task of achieving a good causal explanation. A more complete description and justification of these 10 + 10 cognitive limitations can be found in Saiz (2017, 2020).

Our objective here is to expose the features of our cognitive system that must be taken into account conceptually and applicably in CT, but again, we do not see that this is considered, at least we are not aware of it. If CT aspires to solve real-world problems effectively, it is not enough to prioritize certain key skills that were not there before; we also need to know our enemy’s strengths, that which, even if we proceed well, will distract, distort, or confuse us. We need to know the front and reverse sides of our cognitive machinery, or else we ourselves will be the victims of our fragile mental Achilles heel, which is confirmatory bias. Careful and attentive observation is the foundation on which all causal explanation rests. Technically, making sense of or explaining a problem follows some well-known steps. From these observations, we formulate the corresponding inductive generalizations, i.e., we bet on a first meaningful approximation to reality. Then, we propose our first explanatory hypothesis or our first conclusion from a causal reasoning; in reality, causal reasoning consists of facts plus generalizations. Once we have a proposed causal relationship, we must verify it. This reasoning is not only causal, it is already hypothetical reasoning; simplifying these technical aspects, we will say that hypothetical reasoning is causal reasoning plus verification. By obvious transitivity, observation remains the basis of causal explanation (for more information on these technical aspects, see Govier 2014). We have formulated and integrated this treatment of the causal with the developments that we will present below (see Saiz 2020).

**Figure 3:**
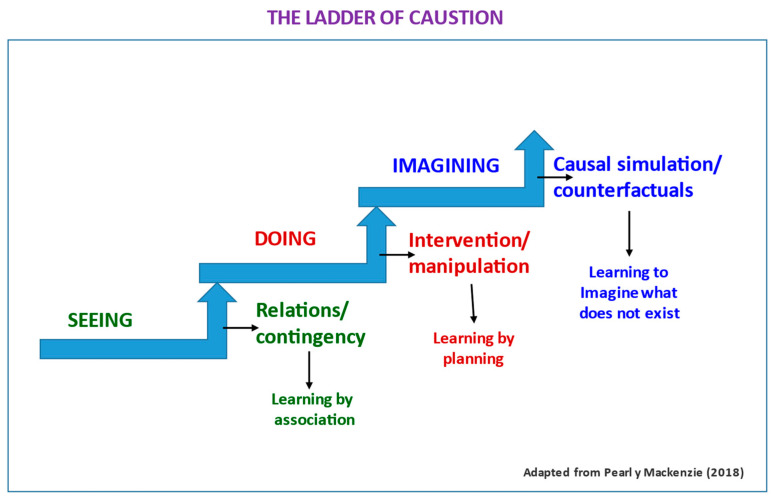
The ladder of causation (adapted from Pearl and Mackenzie 2018).

At a less technical and more descriptive level of causality processes, we must elaborate on part of the above; to do so, we will use Pearl and Mackenzie’s (2018) analogy of the causality ladder (see Figure 3). When we observe, we usually identify relationships between events that, if repeated, lead us to establish generalizations that we then can elaborate and refine to find a cause, or several causes, of a given effect. We can imagine how to proceed in a medical diagnosis—the presence of symptoms or disease combined with the presence of an agent (virus, bacteria…). This would be the first rung or level of the causality ladder, where we can only establish relationships by simple *observation*. Here our learning is only through observation, with which we reach contingency relationships. The next level of the ladder is that of action, *doing*, in which we can manipulate or intervene in reality. In this step, we learn by planning, and it is where we can establish or demonstrate causal relationships because we *can experiment* and modify reality to see what happens given certain conditions. In their magnificent book, *The Book of Why*, the authors point out that only humans are at this second level (an anthropomorphic bias of the authors, of little importance). One only has to see the spectacle of the Okinawan crows manipulating the environment to admit that other living organisms operate on that rung. Kabadayi and Osvath (2017) make a very interesting description of these behaviors.

The third and final level of causality would be that of *imagining*. This is the most interesting step for us because it involves all our sophisticated cognitive machinery, incorporating the consciousness of oneself, of the “I”. It is not within our objectives to deal with questions of animal psychology, so we will not mention exciting studies, again with corvids, on their degree of consciousness with simple experiments using a mirror. We will only mention that in neuroscience, it has been demonstrated that consciousness is located in the medulla oblongata, as this structure is the linking node between the central system and the vegetative system responsible for pain and pleasure, which are the origin of consciousness (see Damasio 2021). For our purposes, the importance of simulating reality, of recreating it internally, lies in the *ability to imagine what has not happened* and what we refer to as *imagination or counterfactual thinking*. Evolutionarily, this capacity seems to be of recent emergence, from only about 40,000 years ago (Pearl and Mackenzie 2018; the most realistic estimate is between 40,000 to 60,000). Being able to imagine what would happen if certain behaviors or events occurred allows us to causally simulate reality without the need for manipulation or experimentation. From an adaptive point of view, this is a colossal qualitative leap. This level of consciousness allows us to learn by imagining what does not exist, that is, to construct causal scenarios that lead us to causal explanations in much less time. This ability enables a level of mental representation and abstraction that has allowed human beings to dominate—and, of course, be able to destroy—their world. See Figure 3, where the causality ladder is shown schematically.

Descending to a more concrete analysis, if in a causal scenario we come to propose A as the cause of effect B, we are saying that, when A happens, B must happen. Alternatively, if B does not happen, neither does A. Simply put, we formulate a conditional proposition: if A happens, then B will happen. What we do is apply to reality the properties of sufficient and necessary condition (SC-NC), of the conditional proposition of the ideal world of deduction. In deduction, we say that “if A, then B”, meaning that if A is true, B will also be true, and if B is not true, A will not be true either. In passing from the ideal world of deduction to the real world, we change the value of truth-falsity to that of presence-absence, nothing more and nothing less. This is the revolution brought to us by the genius John Stuart Mill almost two centuries ago (Mill 1973).

From an applied perspective, pointing out that any explanatory hypothesis is a conditional formulation with its properties allows us to establish a formal correspondence between causality and logical principles (see the technical development in Saiz 2020). If a doctor encounters symptoms or a disease, they will want to find out the cause, so they can cure it. Two examples: (a) a doctor has, for example, 200 patients, some of whom manifest certain symptoms or are ill—some of them have bathed in the same swimming pool, or eaten in the same restaurant, but some are ill and others are not; (b) in a hospital, a patient is admitted to the emergency room with multiple health problems (fever, internal bleeding, high blood pressure…). In both cases, the doctors proceed in the same way, formulating explanatory hypotheses, seeing what the symptoms tell them, and finding out what the true cause is to administer the appropriate treatment to cure the patients. The logic does not change, but the way of applying it does. In the first case, we can rule out causes by SC and by NC, but not in the second example, because in this one we can only rule out causes by NC. In practice, it is important to realize this difference because there are problems or situations where we have data on the causes, but in most cases, we do not; we can only guess, as in the second example. The other practical consideration is that the rules for discarding SC and NC can be replaced by two logical principles, which have the advantage of being more easily applied than the rules of SC and NC. We have, for example, a fever (B) and assume an infection (A); say A and B are present, or if A, then B; the patient is given an antibiotic for that bacterium, and the fever may or may not go away. In the first case, we have applied the rule of discarding NC or the logical principle of negation of the consequent. From experience with our students, we have seen that it is easier to employ logical principles than discarding rules (the full description of this procedure can be found in Saiz 2020 and its application and demonstration in Rivas and Saiz 2023).

Reaching a causal explanation requires observation and application of principles on the facts as well as subsequent verification of those principles. Being able to imagine events counterfactually allows us to construct causal scenarios that have not happened but which, if they did happen, would happen just as we imagine them. Being able to test different causal scenarios in this way considerably increases the probability of finding the correct causal explanation, which allows us to provide a complete sense of the events. Having reached this point, we can predict what will happen and see if time proves us right or wrong without cheating. This is what we call *vital verification*, for without this we cannot be sure of our causal conclusions. With our students, we started working on everything related to causal explanation with a real everyday situation (written for didactic purposes), in which a group of friends spend an afternoon at the house of one of them (see the case in Saiz 2020, p. 42). The friend telling the story liked the friend who hosted them, and after the gathering, he came to the conclusion that he had at least the same chances of going out with her as the other two boys at the meeting.

Despite being a simple everyday situation, common and frequent, our students are not able to figure out if the narrator is right or wrong. To help them in their desperation and to help them understand the importance of the last step of the methodology employed, we tell them that, if this story were happening now, and they were in a place where they could see the entrance to the friend’s house, they would have to be able to test their prediction. They would have to see that, of the three boys, the one who will go to the hostess’s house several times is the boy of behavior X in the meeting, while none of the other two will do so. This is what we mean by vital verification, how we must test our predictions of causality, the only way to be sure of them, because there is also no possibility of cheating (for a full description and practical demonstration, see Rivas and Saiz 2023; Saiz 2020).

CT must be able to deal with real-world problems, and for this, it is necessary to prioritize causal explanation and interaction or action to solve. In the review conducted, we see that there has been an important change in the approach to CT to solve problems, but the way to put this into practice has not yet been developed, or not enough, in the sense of solving effectively and producing the desired changes. For this reason, we say that this is a major problem in CT, which must be solved if progress or advancement is to occur. This is our diagnosis of the problem; the treatment or cure is what we are going to expose next—that is, a project of inquiry into the causal relationships that really exist between CT, personal well-being (PB), and training (FO), understood as integration of what seems to us the most important and clearly interdependent. We will refer to this project as the *CT-PB-FO causal model*. In it, we gather everything discussed so far in an integrated manner, with the proposal of the corresponding causal relationships. In Figure 2, we summarized the environmental and personal factors that determine our personal well-being (see Figure 2).

We have previously commented that personal well-being is a fuzzy concept, and we have referred to an extensive treatment of it in the work on happiness by Flanagan et al. (2023). In a study on the instruction of intelligence, Nickerson (2020) asks several questions, such as whether we can teach it and why to instruct. To answer this second question, he refers to national or social and individual well-being. Again, we will not go into sociological considerations because they exceed our objectives. However, the ethical, moral, or civic must be considered within the CT because it would be good for the instruction to achieve, in addition to intelligent people, good and responsible citizens.

Our position on this is that, of course, this is always desirable, but following the Socratic approach, virtue cannot really be taught (Dialogue of Protagoras in Platón 2018^2^). We are not aware of any demonstration that values are learned; rather, what we have seen very often is that you learn what you do, not what you say. Setting a good example seems to us a more appropriate strategy for this purpose. The controversial conclusion that follows from this is whether thinking critically should imply certain ethics, and our clear answer is that it does not. Let us try to be clear. Thinking critically must be about effective problem solving, just that. Our hands are too small to encompass so much. For example, a lawyer defending a drug dealer should try to get him acquitted, and if he succeeds, he will have done his job well, he will surely have thought critically. Regarding ethical or moral issues, the lawyer must take them into account before accepting the case, that is, when he must consider whether or not his convictions prevent him from defending a criminal. If they prevent him from doing so, that is when he must resign the case, but if he accepts, he must go all the way.

We wanted to steal some space from this question because for us it does not enter into consideration when it comes to critical thinking; we are only concerned with knowing the best way for our cognitive system to function. The non-cognitive components that are part of CT are of interest to us to prevent them from interfering with that functioning. For this reason, when we speak of personal well-being, we mean what is desirable for a person, what he or she will strive for and pursue, such as having a good job, good friends, a family that one appreciates and is appreciated by, social integration, respect, quality of life… In short, what each one believes is best for him is what we will call personal well-being (PB), the goal that will always move us. As we have already said, this is the fuel or the force that drives the vehicle, the one that puts the CT to work, or is it the CT that gets that PB? This is the first question that arises, which we will address later. Now, we are interested in specifying that the motivational aspect is part of this broad space of will or incentive, which can move us or not, and leads us to act. Motivation is a concept that is just as elusive as the rest of the mental processes, so we are content here to equate it with desire, will, or interest in achieving a goal. Of course, as can be seen in the literature, motivation and emotion are not easy to separate. The PB, let us not get confused, is a positive emotional state, and the energy to achieve it, the motivation, again, feeling and will. Here lies the origin of what we are, of the consciousness of oneself, of the “I”, and from this *“I”* arises our abilities and skills. From here is where the evolution of our cognitive system takes place, as Damasio titled his book *Feeling & Knowing* (Damasio 2021). As we would say, feeling, thinking, and knowing (pleasure, process, and its product).

However, as we have already seen, our intellectual skills are not ideal because they are not genetically given to us. We only have elementary skills that allow us to adapt and survive; we need to develop these skills to survive the problems of today’s world. We need to learn to think critically because we are not born with this expertise. It is essential to acquire higher skills and avoid their biases and deficiencies. For this, we need ongoing formation (FO), which will enable us to think well and correctly. Now we have the three essential points of view to develop CT: the descriptive, the normative, and the prescriptive (Baron 2024). The first is the limitations of our processing system, the second is the certain causal explanation, and the third is the way to avoid the limitations and achieve the correct judgment, that is, the formation or preparation without possible rest.

**Figure 4:**
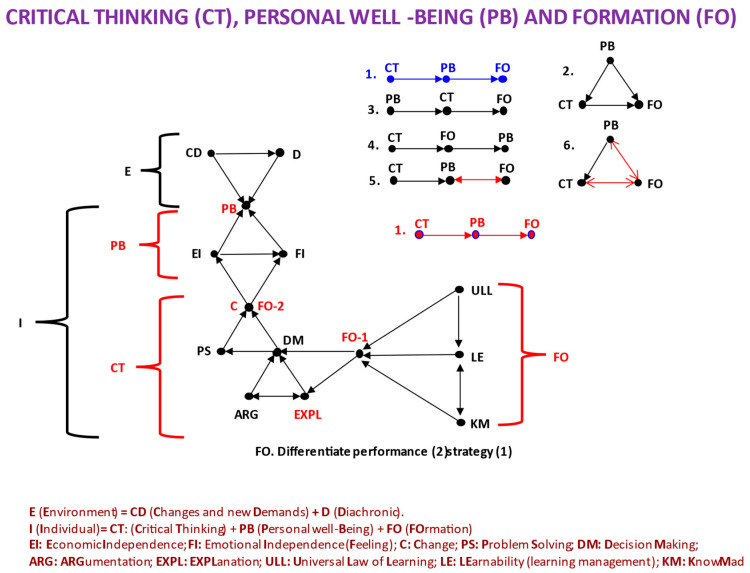
Project of a causal model of the CT-PB-FO.

In the introduction, we set an objective: “…to explore the application of CT to real-world situations and everyday life…”, and now we can propose a way to achieve it, namely, using a causal model of CT-PB-FO. Figure 4 shows such a model, which we will now describe and justify. First of all, it is helpful to understand the two parts of the model that are represented. On the one hand, we have the possible hypotheses of causal relationships that we can imagine (upper right part of Figure 4); on the other hand, we specify the different factors that we must consider in this causal model, the environmental (E) and personal-individual (I). In this sense, what appears as FO in Figure 2 is what we formulate in Figure 4 as FO-2, that is, the result or performance of the formation. FO-1 is everything we have described as formation or acquisition strategies. We will see that this difference is important.

We continue with the model in Figure 4 as a proposal to improve CT to respond to real-world problems. However, we offer an open causal proposal since there are not yet sufficient data to be able to rule out some causal relationships and propose others. The first and simplest thing is to know what causal relationships are established between CT-PB-FO. Are these relationships unique, or are there several? In Figure 4, we have represented six possible types of causal relationships that, from the existing knowledge in the field, seem acceptable (see upper right part of Figure 4). We have highlighted the first one as the most conceptually convincing, but it is a bet; we have no data to prove that it is the correct causal relationship. However, causal sequences 3 and 4 also compete with 2 in a very meaningful way. In contrast, if we look at sequences 2, 5, and 6, we see that the causal relationships are not straightforward. Technically, these relationships are confounded, and additional variable measures would be needed to achieve “de-confusion” (see Pearl and Mackenzie 2018). Sequence 5 shows another type of difficulty, as it shows an arrow joining two nodes bidirectionally. When this kind of linkage between nodes is indicated, our causal ignorance is manifested because we cannot state what causes what. Therefore, before moving on to verification, a solely unidirectional relationship, such as those in 1, 3, and 4, must be justified. Recall that this system of graphs used as a representation of causal models is a logical system that Judea Pearl employs to represent what he calls the “new science of causality”; these diagrams are simple and clear and allow us to specify all imaginable causal relationships as well as all their complications. This system is the one we are employing for our proposal. Therefore, the entire conceptual foundation is based on Pearl (2009; Pearl and Mackenzie 2018) and the measurement procedures supported by Bayesian networks in Pearl et al. (2016) since this is a manual written to facilitate the calculation of this kind of conditional probability equation. It is beyond our scope to detail the different types of calculations that must be taken into account before being able to affirm that a causal relationship exists, in our case, between three nodes (CT-PB-FO or other combinations). To arrive at the establishment of these causal relationships, bidirectional relationships between variables must be eliminated by identifying the correct mediators or confounding or lurking variables and achieving de-confusion by means of the back-door criterion. Probability measures must take into account all these complications, which are very well described in Pearl and Mackenzie (2018).

Does CT contribute to PB and PB to performance (FO-2)? The question is simple, although still difficult to answer. However, this is what it is all about, to know what causes what, to proceed from an applied point of view. As we said, logically, the first thing to do is to demonstrate which causal model works. To do this, we then need to justify the rest of the conditioning or causal factors, which are specified in the rest of Figure 4. In the rest of the figure, we find three blocks of components: environmental, personal, and formation strategies (FO-1). Regarding personal formation for the development of CT, we have previously proposed a system consisting of knowledge acquisition based on explanation, solving real problems and producing changes, a lot of inter- and intradomain practice, and a lot of individual autonomous work. This is the way to avoid the negative consequences of ULL, to optimize learning management (learnability; LE), and to increase horizontal or transversal training (knowmad; KM), currently demanded (see Figure 1), to be able to face the problems of daily life (see in Saiz et al. 2020). We have incorporated this way of developing or improving CT into in an instructional methodology that we have been able to verify recently, with very robust data, which is part of the support for the model proposed in Figure 4, specifically, the causal relationships between the CT and FO brackets (Rivas and Saiz 2023).

The next block of the causal model in Figure 4 refers to the personal components, specifically, to fundamental CT skills. As can be seen in the model, the formation is essentially focused on causal explanation, which, in turn, determines the process of decision making and problem solving. Deciding and solving are processes that are difficult to distinguish beyond the fact that in the former, the options are available, while in the latter, we must discover or create them, since in everything else they are indistinguishable (Saiz 2020). We would say that CT is to explain (EXPL), decide (DM), and solve (PS) to produce a change or achieve a goal. The relationship between the cognitive components should be in this way, the EXPL (the pre-decisional) determining DM and PS (the post-decisional). Argumentation (ARG) contributes to enhancing EXP and DM. In the instructional program that we have developed and verified, we work with this model, applied to the solution of personal and professional problems (see in Rivas and Saiz 2023).

In this block of the model, outcome, change, or academic or professional performance is fundamental. If you do not see achievement, you do not improve your CT skills. In the years that we have been working with our students on this CT development, we have found that without visualizing some kind of change, competencies are not consolidated. In our research, we have seen that what really makes CT improve is when one sees it improve (Rivas and Saiz 2023; Saiz and Rivas 2011, 2012, 2016; Saiz et al. 2015). Our data show us that motivation is highly overrated. Alfred N. Whitehead was right when he said: ”There can be no mental development without interest. Interest is the *sine qua non* for attention and apprehension. You may endeavour to excite interest by means of birch rods, or you may coax it by the incitement of pleasurable activity. But without interest there will be no progress”(Whitehead 1967, p. 31). After all these years of applied research, and with the changes of the current times, our skepticism has increased considerably in the sense of seeing the few changes that are obtained from motivation. Either the interest, not just the utility, is in us, or if it is not there, it is not going to emerge. Increasingly, our work in instruction is focused on the acquisition of fundamental skills applied to real problems in which consequences, positive or negative, are observed. This can be said to work well (Rivas and Saiz 2023).

After having posited the causal relationships between FO strategies and fundamental CT skills, on the one hand, and their consequences in terms of performance and achievement, on the other, we must move on to the causal relationships of CT with personal well-being (PB). Recall that we mentioned earlier that personal growth and maturity depended on achieving the double objective marked by the dual nature of the human being (biological and social), namely, economic (EI) and emotional independence (FI). Without these two objectives, a person cannot function well in any area. Now, the adequate development of CT skills will be the fundamental tool to reach this double maturity, as long as changes or results are achieved in the solution of daily problems. Ineffectiveness always prevents PB, but the opposite enhances and stabilizes it. As we can see, despite the large number of nodes and relationships in this causal model, in the end, it all continues to be summarized in the triad mentioned in Figure 2, CT-PB-FO, although the precise causal order is still uncertain, as it requires data to be able to establish some and discard others. As we have pointed out in the PB section, we have few data (of the self-report type) that do not allow us to prove the proposed causal relationships independently. In this proposal, we only need to mention the environmental causes or determinants and how to quantify all this.

Changes and new demands, as we have already seen, influence PB insofar as we must continue to face real problems with new resources and learning strategies. The attention capacity must be recovered, and the ability to observe correctly must be developed. Screens and leisure must be controlled from CT competencies, and autonomy or personal initiative (as in the formation) and responsibility or a greater awareness that behaviors have consequences must be increased. On the other hand, the life cycle, or the diachronic dimension, has a much greater influence than we think, especially in the critical period of transition to adulthood; this is where cognitive and social competencies are consolidated in a few years to achieve the economic and emotional independence essential for PB. The diachronic dimension, and especially its critical period, is one of the most neglected in personal formation (Saiz 2021; Saiz and Rivas 2020).

Let’s say that the environmental factors mentioned above always have a negative influence if we do not adapt or take advantage of these changes and demands. The ULL illustrates it very well: we must change in order not to stagnate and change much more to progress. Technologically, the current times offer extraordinary resources, unthinkable only less than two decades ago; however, at the same time, they are like a spider’s web that envelops us and can immobilize us. The information available reduces exponentially the time of consultation for searches, which previously required days or months, but this ease can lead us to a huge ocean in which we end up not finding what we are looking for or finding an impostor substitute. On the one hand, all this requires good cognitive skills to acquire the knowledge that allows us to solve everyday problems, and on the other hand, non-cognitive skills, such as initiative or autonomy, enable us to apply these skills while avoiding our own limitations, deficiencies, or unconscious influences. One may have learned to analyze, for example, arguments correctly, but must, at the same time, develop sufficient sensitivity to detect the false solidity of a good fallacy. Many of our mistakes happen because we are not aware of them. We can only avoid them with familiarity, that is, with practice. Falls or missteps, for example, when riding a bicycle, are avoided with practice. There is no theory to apply to this; as an analogy, it only serves to bring to consciousness the corresponding skills with practice and application.

The important discoveries in neuroscience, the crucial contributions and help that AI is beginning to offer, the development of applications (“apps”) to perform a multitude of tasks, the sophisticated mathematical models that save hundreds of experiments in fields such as cell biology, or the new methods of demonstrating causality, such as the one we are dealing with, should enrich the conception and research of CT. We propose a new or improved conception of CT, a form of logical specification of that conception, and the incorporation of a new mode of demonstration or hypothesis testing. The new mode of demonstration, as Judea Pearl himself tells us, consists of performing greater conceptual precision using logical systems such as graphs and performing node-to-node calculations using Bayes’ or conditional probability theorems (Pearl and Mackenzie 2018). This system of demonstrating causality began to be used in the 1930s, and it was a great researcher, Barbara Burks, who began to use “path diagrams” to study causality, in this case, to study the heritability–environment determination of intelligence. At that time, this brilliant and ill-fated^3^ social science researcher demonstrated clear causal relationships with these logical diagrams and the calculations that these diagrams demanded. Obviously, this approach went against the prevailing statistics, in which the word *cause* was taboo, for great figures such as Karl Pearson and the researchers who worked guided by his conception (see Pearl and Mackenzie 2018).

This influence and rejection of the concept of causality, and replacing it only with that of correlation, meant that these developments of causality models and their measures did not become widespread until half a century later (see in Pearl and Mackenzie 2018). Today, this approach is conspicuous by its absence in the social sciences. It has not been so in medicine because of the importance here of demonstrating causal relationships in the treatment and prevention of disease. Just as an example, until the end of the 1980s, it was not possible to demonstrate the causal relationship between smoking and cancer. This was not demonstrated experimentally—it is not possible—but through causal models, such as those extensively developed by Pearl (see again in Pearl and Mackenzie 2018). Is it time to recover the tradition of Barbara Burks in the social sciences? We believe it is. To do so, it is necessary, as Pearl suggests, to move away from the approach of the great statistician Ronald Fisher, from randomized controlled experiments and their statistical significance. “If our conception of causal effects had anything to do with randomized experiments, the latter would have been invented 500 years before Fisher” (Pearl and Mackenzie 2018, p. 62). This conception of causality forces us to specify each determinant and to perform individual node-by-node calculations using simple conditional probability formulas (Pearl et al. 2016). This is what we propose to apply to CT.

The causal model we propose in Figure 4 demands the specification of each causal relationship between factors or variables, the identification of confounding relationships and of “back doors”, to block from the latter the influence of confounding variables on the causal relationship (see Pearl and Mackenzie 2018; we cannot detail all these technical aspects of the confounding or back door variables, as they are beyond the scope of our work). Achieving this conceptual specification requires a detailed deduction of possible relationships and the elimination of inconsistent ones. Once this work of logical formulation using graph diagrams is completed, we move on to the measurement of those relationships at each of the arrows or junctions between nodes. Once we have these data, we can perform the probability calculations of the whole causal model, with the corresponding formulas, for the causality, confusion, and “back door” relationships. Let us not forget that the double-arrow node connections are conceptually imprecise and must therefore be removed before any measurements can be made. It falls outside our objectives to go into further conceptual and computational details (see in Pearl 2009; Pearl et al. 2016; Pearl and Mackenzie 2018). The model we offer as a proposal for CT development and its application to real-world problems is an incipient proposal that requires justifying some causal relationships and ruling out others and then being able to measure them and demonstrate that those are the relationships and not others. The purpose here is not to offer a developed causal model but to show how it can be developed with this new treatment of causality. Therefore, the objective of this work is to offer an open causal proposal, since there are not yet sufficient data to be able to rule out some causal relationships and propose others. As mentioned above, we only have data for the relationships included in the CT and FO brackets in Figure 4 and insufficient data for the relationships within the PB and E brackets. We have only been working with this model for a short time, so we need more studies to narrow down the possible causal relationships between the different factors involved in the model. At the same time, these data will allow us to eliminate confounding and circular variables that can be proposed but cannot be debugged.

## 7. Discussion and Implications

Throughout this paper, we have proposed a research project that provides a solution to a substantial CT problem and have presented a causal model that seeks to better articulate fundamental CT skills to effectively solve the problems of the real world. This project should promise a relevant contribution to the field of CT, and we believe it makes good on the promise. When we talked about the CT problem, as we have been justifying it throughout this work, we pointed out a limitation, a difficulty regarding which process is responsible or the most important, when it comes to solving everyday problems or achieving our goals. We indicated that the fundamental mechanism cannot be other than the causal explanation because otherwise, it would not be possible to solve the problems of the real world well—because this imposes interaction, and action, and for this we need causality--. We saw that this is not in the foundation of CT, or not as it should be. In fact, in general, we justify a kind of ignorance or difficulty in this field of research.

The justification of a problem consists of making it clear that it exists and that it can be solved; and the solution is always a proposed response to the problem. This answer must always be given through a research project: a limitation or lack of knowledge (the problem), an explanation or solution (the proposal), and the demonstration or verification that it works (verification). This is how we understand a research project, as a relevant and original solution proposal, which has not been offered until now. The importance and novelty of the project is what makes it a research project and not something else since it has to be a contribution to the field of knowledge; to be considered a contribution, it has to be demonstrated or verified with facts (see Kerlinger 1986). Once the project has been tested, it is no longer a project but a reality. Our project has become a reality only in part. The rest must be the subject of future research.

The proposal developed throughout this work has been empirically tested in that part made reality, in particular, regarding the relevance of causality in terms of solving everyday problems and in what has to do with formation or instructional strategies for the development of CT (Rivas and Saiz 2023; Saiz and Rivas 2016). However, as this project is also a proposal for a causal model linking CT-PB-FO, many causal relationships remain to be specified and tested, something we are currently developing. In this model, a new way of formalizing causality is employed using graph diagrams, which impose different computational procedures for verification. The proposed general model only points to possible causal relationships, which must be empirically discarded to establish the true determinants to have a precise conceptual system that allows the subsequent verification of their relationships node by node.

Thus, our proposal offers relevant conceptual solutions that can make CT an effective tool for solving real-world problems. The incorporation of causality as a fundamental competence forces us to give greater prominence to post-decisional competences (action) to produce the changes that will make our objectives a reality. Continuous formation in CT development allows us to achieve personal well-being, which is essential to achieve self-reliance and personal stability or maturity, which is also expected to make us responsible citizens committed to the common good. Obviously, there are still important limitations to this project, such as the problems of measuring some of the supposedly causal factors or variables.

In this sense, it is important to avoid self-report-type instruments and look for tests that are tasks or problems to be solved. In this respect, there are measures of critical thinking whose items are everyday problems or situations to be solved. A pioneering test in this direction is that of Diane Halpern (Halpern 2018). Inspired by a part of the approaches of this test, we validated another one that adds the task analysis methodology to elucidate what process is being used to solve each item or problem posed (Saiz and Rivas 2008; Rivas and Saiz 2012; Saiz et al. 2021; Rivas et al. 2023a). Despite the availability of some measures of CT skills, we still lack several that would allow us to quantify causal relationships with instruments that are problems or performance tasks, not self-reports. Quantification is essential in verification procedures, so other ways of accurately measuring the rest of the variables in the proposed causal model, which have not yet been tested, must be developed. For this reason, the project must await the availability of behavioral measurement tools for the rest of the variables that have not yet been evaluated before it can be fully implemented. For example, one of the most problematic quantifications is personal well-being. It is not easy to obtain objective indicators of the vivencial and, as we have already said, self-reports or personal assessment of emotional states are not useful; at least, they are not when we seek to demonstrate causal relationships. At the moment, we have some incipient advances in this respect, as we have already mentioned above, which go beyond the scope of the present work.

## Data Availability

Not applicable.

## Notes

1 Dupin is the protagonist of some of Allan Poe’s famous stories, such as *The Murders in the Rue Morgue*.

2 The sophists understood virtue as equivalent to social success, but for Socrates, virtue was the care of the soul; for him, with logos we attain knowledge; with it, virtue; and with it, happiness.

3 Barbara Burks ended up jumping off one of the bridges in New York because her colleagues closed the doors of all the universities where she applied. They never recognized her work, nor that she was right in her studies, besides making her life impossible during her doctorate.
